# Exploring the impact of housing routine on lying behavior in horses measured with triaxial accelerometer

**DOI:** 10.3389/fvets.2025.1572051

**Published:** 2025-05-12

**Authors:** Elena Gobbo, Chiara Maccario, Manja Zupan Šemrov, Marco Bovo, Elie Atallah, Michela Minero, Emanuela Dalla Costa

**Affiliations:** ^1^Department of Animal Science, Biotechnical Faculty, University of Ljubljana, Domžale, Slovenia; ^2^Department of Veterinary Medicine and Animal Sciences, Università degli Studi di Milano, Lodi, Italy; ^3^Department of Agricultural and Food Sciences, University of Bologna, Bologna, Italy

**Keywords:** horse, lying, accelerometer, positive welfare, animal-based measure

## Abstract

**Introduction:**

Methods to assess the positive behavior of horses in relation to their environment can be used to provide information to enhance animal welfare. One of the most important experiences that can be observed in mammals is sleep, a universal behavior relevant for the welfare of all species. To achieve paradoxical sleep, horses must lie down in lateral recumbency for a sufficient time, but they only do so when feeling safe and comfortable. Recently, technological tools like accelerometers have opened the possibility of non-invasive continuous monitoring of lying behavior, thus implementing the way we assess equine behavior in relation to their management and environment.

**Methods:**

The aim of this study was to investigate whether a sudden change in housing routine affects lying behavior in horses. In 10 riding school horses, lying behavior was continuously monitored using triaxial accelerometers for two separate 5-day periods, each under a different housing routine (i.e., ordinary: in a paddock in small groups; modified: in single boxes).

**Results:**

The results show no statistical differences in the total daily duration of lying behavior between ordinary (25.19 ± 21.81 min) and modified (23.16 ± 20.05 min) housing routines. However, in the ordinary housing routine, when horses were kept outdoors in groups of varying sizes, larger groups exhibited synchronized lying behavior, with longer lying bouts, while smaller groups lay down more frequently throughout the day.

**Discussion:**

The results show that sudden change in housing routine does not have a significant effect on lying behavior, while group size appears to be an important factor for behavioral synchronization. However, the small sample size, the single location, and mixed-age and sex population may have influenced the findings. Accelerometers were shown to be beneficial for monitoring natural behaviors such as lying and thus inferring information about equine behavior in relation to daily routine management.

## Introduction

Horse (*Equus caballus*) is a versatile species that can be kept as companion animals, for sport, leisure, and for production. The adaptability of this species to different contexts worldwide has led to the growth of the equine industry, with approximately 58.6 million horses registered by 2021 (1). This growth has simultaneously raised the public concern for their welfare (2), with housing and management playing a crucial role (3), as they can influence physical exercise and the ability to lie down due to reduced space, social interaction and perceived safety through group vigilance due to isolation; as well as lead to a lack of comfort (presence of artificial light, bedding type and depth, temperature and humidity); and hypo stimulation, which lead to lethargy and mental fatigue (3). In this sense, resilience is another important concept related to welfare which refers to the degree to which an animal's behavior and physiology are affected by stressors or challenges (e.g., change of housing conditions). Positive experiences can instead enhance resilience, enabling individuals to better cope with challenging situations (3). Over the past decade, there has been an increasing interest in finding methods to assess the positive experiences (i.e., condition or interaction that promotes positive states, enhances survival and fitness, or serves as a reward an animal will work for) and welfare of horses in response to their management and environment. Several studies have shown that healthy horses living in a natural environment dedicate a large part of their time to all the behaviors intended to keep the animal alive and healthy, also defined as “maintenance behaviors” (4). Among these, sleep is particularly important as it is essential for both physiological (e.g., high caloric ingestion without weight gain, reduction in anabolic hormones) and cognitive functions (e.g., regulation of neuronal functioning during memory storage and consolidation and cerebral metabolism within the prefrontal cortex, responsible for judgment and decision making) (5–7).

Sleep is defined as a maintained state of quiescence characterized by relative inactivity, loss of consciousness, and/or increased threshold of arousal to environmental stimuli. It is regulated by circadian rhythms, which organize timing over 24 h, and by homeostatic mechanisms, which determine the amount of sleep required for each species (5). Horses exhibit a polyphasic sleep pattern, distributed across 5–7 shorter episodes, with most sleep occurring at night, particularly between 12 a.m. and 4 a.m. (8). To achieve paradoxical sleep, horses need to lie down for sufficient time in recumbency (9). However, as prey animals, horses only lie down when they feel comfortable and safe (10, 11). Consequently, measuring lying behavior can be a valuable starting point for monitoring natural maintenance behavior, thus inferring about positive welfare (11).

Variations in management can significantly influence the amount of time a horse spends in decubitus, which in turn affects its time in Rapid Eye Movement (REM) sleep. Studies have shown that lying behavior of the horse depends on biological characteristics, such as age (i.e., increased in young animals) (12), sex (12, 13) and breed (11). The latter is affected as well by social factors, such as herd size (14, 15), hierarchical position (12, 16), health conditions such as joint damage and chronic orthopedic disease (11, 17), body condition score (11), seasonal changes (12), and housing factors, such as light conditions (10), auditory stimuli (e.g., presence of music) (8), litter height and type (10, 18, 19), and dimensions of the resting area (12, 16, 20, 21). Examining the duration of lying behavior is a valuable tool for assessing positive experiences, particularly, the continuous monitoring of subjects over several days. However, both continuous real-time monitoring and analysis of 24-h video recordings are challenging due to time constraints and accuracy (22). For this reason, precision tools for monitoring and tracking lying behavior can provide greater objectivity in assessing an individual's behavior by facilitating accurate analysis and quantification of the time spent in different activities (22, 23). Accelerometers have been reported to accurately measure the amount of time the horse spends in decubitus, taking advantage of the principle of the absence of gravitational force in the horizontal position of the instrument itself (22, 23).

One of the most common phenomena in horses that can disrupt sleep is a sudden change in the housing condition. Horse housing can be often modified to accommodate individual needs (e.g., box housing due to injuries), for welfare improvements (e.g., paddock/group housing during the dry season), and work needs (e.g., competitions and training schedules). This can negatively affect horse welfare by disrupting established routines and causing stress, especially if the basic needs in the changed housing are not met (e.g., transition from spacious group pasture to restrictive individual boxes). The aim of this study was to investigate whether a sudden change in housing routine affects animal-based measures and lying behavior in school riding horses. Additionally, since horses were housed in groups of varying sizes, we examined the impact of social groups on lying behavior and synchronization.

## Materials and methods

### Ethical approval

All procedures were approved by the Committee for the Welfare of Animals for Experimental Purposes of the Veterinary Faculty of the University of Ljubljana (033-5/2024-5).

### Horses, management and housing routines

The study was conducted at the Educational Research Center for Horse Breeding Krumperk in Gorjuša, Slovenia, during the summer season, from June to September 2024. The experimental period was characterized by stable climatic conditions. The study involved 10 healthy Lipizzan horses (6 mares and 4 geldings) aged 10 to 20 years (mean age 14.8 ± 3.7 years). All horses were trained and ridden using classical English riding methods and were regularly used in a children's riding school. The horses were housed in standard individual boxes (3.0 × 3.5 m^2^) with wood shavings as bedding and visual access to other conspecifics. By assessing the horses' body size, we determined that the boxes were adequately sized to allow all horses to lie down in lateral recumbency and get up without restriction. They had *ad libitum* access to hay and fresh water, and they were additionally fed a barley-oat mixture. The horses also had access to four outdoor grazing paddocks, each measuring 0.5 hectares. These paddocks provided water and grazing opportunities and did not include a specific resting area. One paddock housed four horses, another housed three, the third housed two, and the last held a single horse (see Supplementary Table S2). The group compositions were the same in both treatments, and they had been constant for many years prior to the study and were deliberately structured to include horses that exhibited mutual social compatibility. Horses had visual access to at least one other paddock. During weekdays, the selected horses participated in riding school activities between 17:00 and 20:00, with each horse engaged in ridden work for a maximum of 2 h per day.

**Figure 1:**
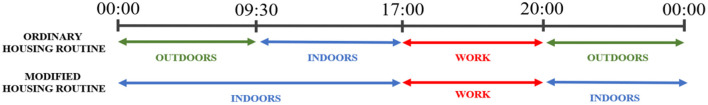
Different housing routines of horses during the working days.

Data were collected twice from the same horses under two different housing routines: ordinary housing routine and modified housing routine (Figure 1). During ordinary housing routine, all horses were housed in individual boxes during the daytime hours of higher temperatures (09:30–17:00) and allowed access to outdoor confined paddocks (either individually or in groups) overnight. On non-working days, they remained in single boxes from 09:30 to 20:00 and were turned out overnight (20:00–09:30). Under the modified housing routine, horses were kept stabled in individual boxes without pasture access and were let outside the box only during riding sessions. On non-working days, they remained continuously housed in single boxes without outdoor access. Due to the summer season, the horses followed their ordinary housing routine. Next, to create a sudden change, they transitioned to a modified housing routine for 1 week. After 1 month, all horses were re-evaluated under the ordinary housing conditions.

### Data collection of lying behavior and data processing

**Figure 2:**
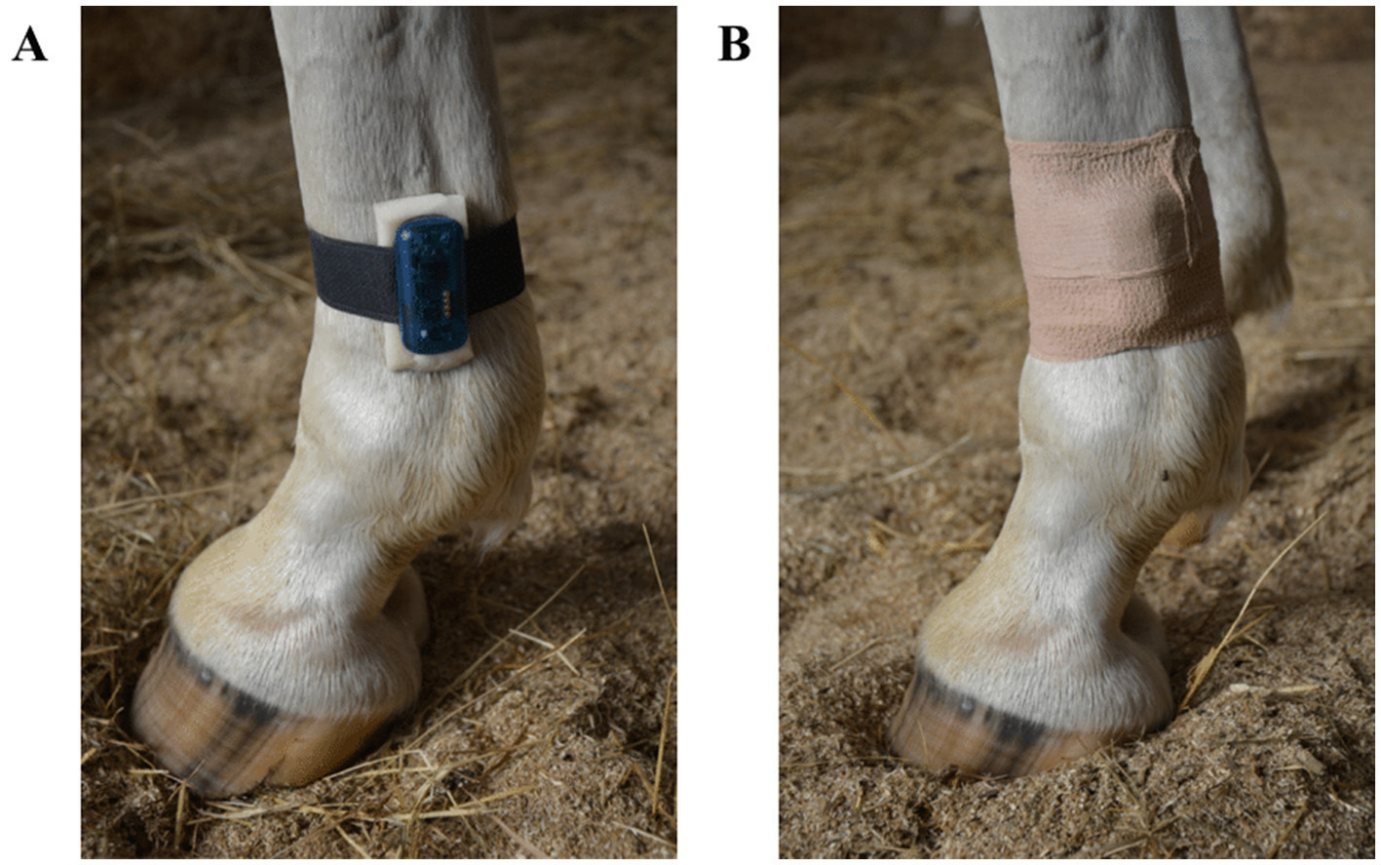
Positioning and application of a triaxial accelerometer during data collection of lying behavior. **(A)** Foam padding placed beneath the device to prevent pressure on the bone; **(B)** Wrapping of the device with an elastic band to keep it safely and firmly in place.

Lying behavior, defined as a posture in which a horse adopts either lateral or sternal recumbency (12), was continuously recorded for 5 days (120 h per horse) using MSR145 triaxial accelerometers (MSR Electronics GmbH, Seuzach, Switzerland). The MSR145 was set at a sampling frequency of 1 Hz. According to the literature, this sensor has been validated for estimating lying behavior in horses (12). For each horse under each housing routine, data was collected over 3 working days and 2 non-working days. The accelerometer was attached to the metacarpal bone of the left forelimb using a Velcro strap (Figure 2). To prevent pressure on the bone and minimize the risk of abrasions, foam padding was placed beneath the device (Figure 2A). Additionally, the sensor was secured with an elastic band to protect it from damage and ensure stability (Figure 2B).

At the end of each monitoring period the accelerometer data was downloaded from the MSR software to a.csv file. Then, the raw data time series were analyzed using MATLAB R2024b. The calculation of the lying position has been estimated starting from the assumption that the accelerometer was positioned by orienting the Y-axis parallel to the limb (vertical), the X-axis in the cranial direction and the Z axis (lateral) perpendicular to the limb. Before processing the data, preliminary numerical check and data-cleaning were carried out. A first check was carried out with the aim of evaluating the correct positioning of the accelerometer on the horse's limb during the monitoring period. Then, from the analyses of the modification of the acceleration values along the Y-axis it has been possible to identify the moment associated with vertical or horizontal position of the limb. When the limb was identified in horizontal position it was possible to assume the horse in lying position (12). Then, for each horse, estimates of hourly and daily lying time were obtained by summing the decubitus measurements per hour and per day, respectively. Counts and duration of lying bouts were also assessed. In the count of the number of lying bouts, a threshold of minimum duration equal to 2 min has been applied (12).

### Animal-based measures assessment

On the final day of recordings for each housing routine, animal-based measures (ABMs) from the first level of the AWIN Welfare assessment protocol for horses (24) were collected by a trained observer. For a detailed description of the assessment methodology of each ABM refer to the AWIN welfare assessment protocol for horses (24). Each ABM was considered as binary variable (appropriate/non-appropriate): the scoring system used for each evaluated ABM is presented in Supplementary Table S1.

### Statistical analysis

All statistical analyses were conducted using SPSS 28 (SPSS Inc., Chicago, USA). Differences were considered statistically significant if p < 0.05. A Generalized Linear Mixed Model (GLMM) was used to evaluate the effects of housing condition, day of the week (working and non-working days), group size (≤ 2 horses or >2 horses), and the interactions, housing condition × day of the week and housing condition × group size, on mean lying duration. To assess lying bouts frequency and lying bouts duration, the housing condition was included as the main fixed effect. Horse identity was included as a random effect in all models to account for repeated measures. Mean lying duration and lying bouts duration (continuous data) were modeled using a Gamma distribution with a log link, while lying bouts frequency (count data) was modeled using a Poisson distribution with a log link. Model selection was guided by biological relevance and comparison of model fit using the Akaike Information Criterion (AIC). Model stability and estimate reliability were assessed by inspecting standard errors and confidence intervals of the fixed effects. Due to the unbalanced sample size across group size categories (*n* = 3 for ≤ 2 horses; n = 6 for >2 horses), interaction effects involving group size are interpreted with caution. Results are reported as estimated marginal means ± standard error of the mean (SEM).

For animal-based measures analysis, the proportion of appropriate scores for each welfare indicator (e.g., percentage of horses with absence of lesions or showing no avoidance when approached by an unknown human) was calculated in each housing routine. A Chi-square test was used to investigate possible differences in the percentage of each ABM between the two housing routines.

## Results

### Animal-based measures (ABMs)

**Figure 3:**
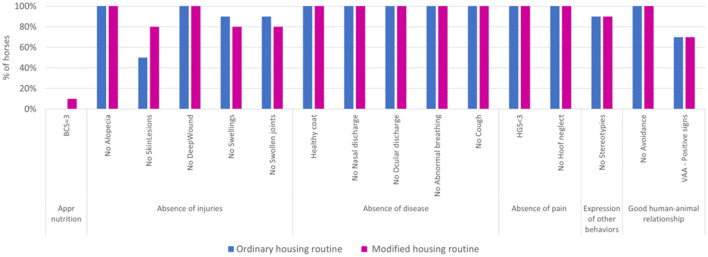
Bar plot reporting the prevalence (%) of horses with no alterations in ABMs in the two housing routines.

The percentage of horses with appropriate scores of different animal-based measures in the two housing routines is reported in Figure 3. No differences were found between the two housing routines (Chi-square test, *p* > 0.05).

### Lying behavior

**Figure 4:**
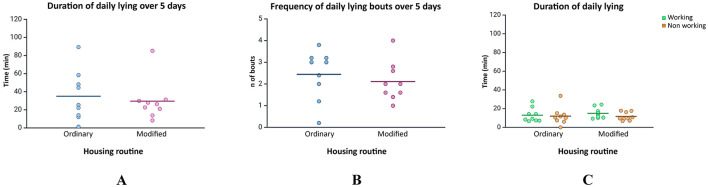
Graphs reporting the lying behavior in ordinary and modified housing routines where each dot represents a subject, while the line is the mean value. **(A)** Mean daily lying duration (minutes) over 5 days; **(B)** Mean daily frequency of lying bouts over 5 days; **(C)** Mean daily lying duration (minutes) in the working days and in non-working days.

One of the horses never showed lying behavior during the data collection and was therefore excluded from the analysis. Although not statistically significant, the mean daily lying duration in the ordinary housing routine was longer than in the modified housing routine (*p* = 0.809; β = −0.165, *t* = −0.426), with 25.19 ± 21.81 min and 23.16 ± 20.05 min, respectively (Figure 4A). Considering lying bouts (Figure 4B), no statistically significant differences were found between housing conditions (*p* = 0.788): in the ordinary routine, horses lie down 2.67 ± 1.69 times per day, and the mean duration for each lying bout was 10.14 ± 6.24 min. While in modified routine, horses lie down 1.83 ± 1.56 times for a mean lying bout duration of 10.85 ± 6.68 minutes. Comparing weekdays, lying duration was slightly higher on working days (12.42 ± 4.97 min) than on non-working days (10.95 ± 4.39 min), though this difference was not statistically significant (*p* = 0.278; β = 0.001, *t* = 0.006). Group size comparison results showed that horses in smaller groups (≤ 2 horses) had a mean lying duration of 15.78 ± 14.11 min, compared to 36.99 ± 31.76 min in larger groups (>2 horses), with no statistically significant difference (*p* = 0.318; β = −0.933, *t* = −1.702). No significant interaction was found between housing condition and day of the week (*p* = 0.223; β = 0.250, *t* = 1.218), with the modified routine having a non-significantly longer daily lying duration (*p* = 0.162) during the working days (13.51 ± 5.50 min) than during non-working days (10.52 ± 4.28 min). Similarly, no statistical differences were found between working (11.41 ± 4.65 min) and non-working (11.40 ± 4.68 min) days during the ordinary routine (*p* = 0.995; Figure 4C). In addition, the interaction between housing condition and group size was not significant (*p* = 0.813; β = 0.161, *t* = 0.241), with horses housed in modified housing routine, having a lying duration of 15.75 ± 14.73 min in smaller groups (≤ 2 horses) and 34.06 ± 29.98 min in larger groups (>2 horses; *p* = 0.371). As in ordinary housing routine, lying duration was 15.80 ± 14.78 min in smaller groups and 40.17 ± 35.35 min in larger groups (*p* = 0.341).

**Figure 5:**
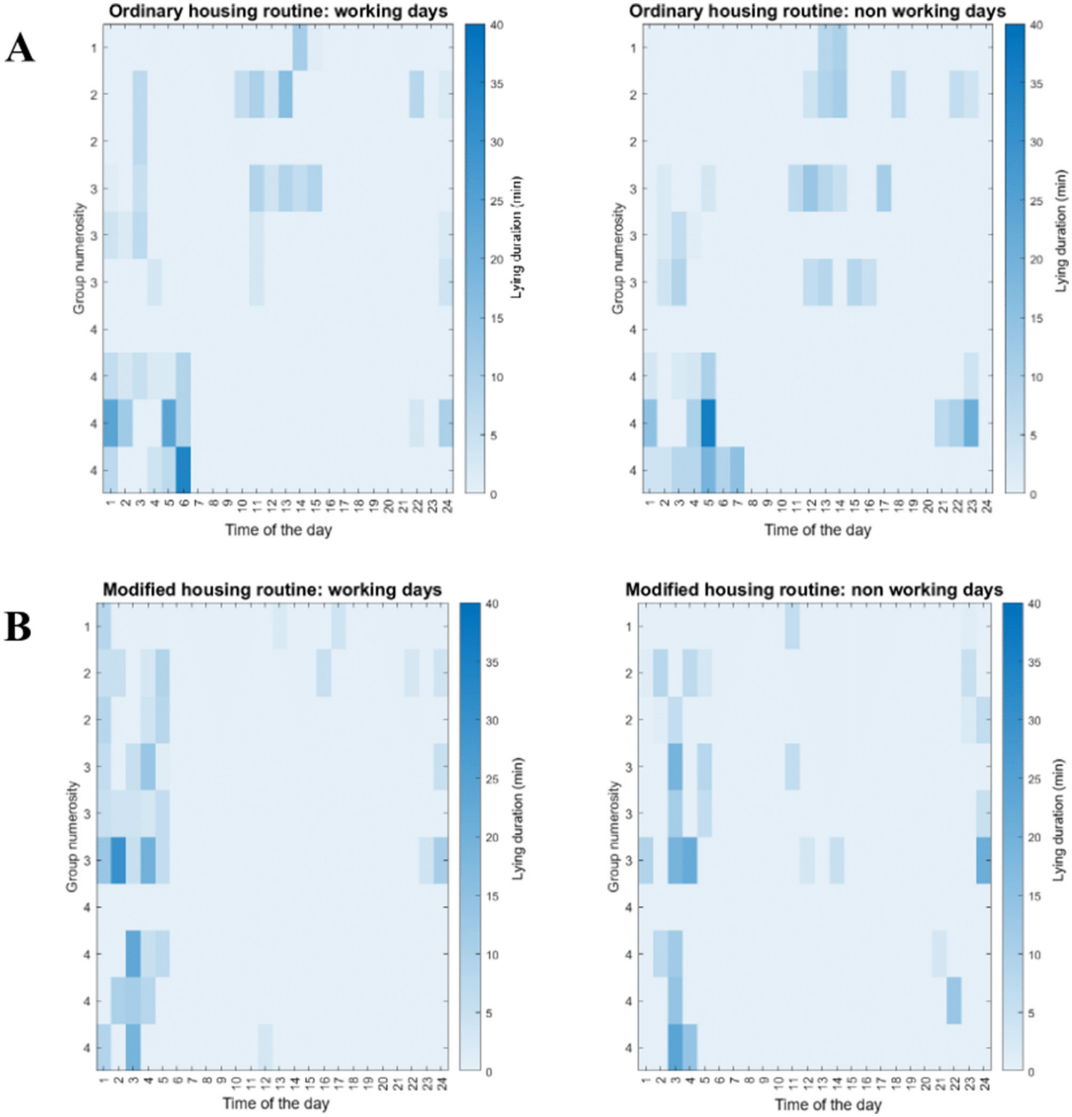
Heatmaps reporting the lying behavior synchronization within each horse group in the ordinary and modified housing routines during working and non-working days. The color represents the mean duration in minutes (0–40 minutes per hour) of lying behavior over the 24 h (time of the day) in working and non-working days and for groups of different numerosity (from 1 to 4 animals). **(A)** Ordinary housing routine; **(B)** Modified housing routine.

The assessment of lying behavior synchronization showed that when kept in groups of 3 and 4 horses, animals seem to lie down at the same hours of the day (Figure 5). In the ordinary routine horses in the same group tended to lie down at the same hours of the night and day (between 22:00 and 6:00; Figure 5A). On both working and non-working days, horses from paired and individual housing frequently lay down during the day, while, in the modified routine housing, all the horses preferred to lie down between 23:00 and 5:00 on both working and non-working days (Figure 5B).

## Discussion

Our results showed no significant differences between the two housing routines in the percentage of appropriate scores of ABMs, confirming that horses were healthy and in an overall good welfare condition in both routines. In terms of lying behavior, horses in the ordinary housing routine appeared to have a longer daily lying duration compared to those in the modified routine, although this difference was not statistically significant. During the ordinary housing routine, when horses were kept outdoors in groups of varying sizes, larger groups exhibited synchronized lying behavior, with longer lying bouts, while smaller groups lay down more frequently throughout the day. While horses seem to express natural behavior in both housing routines for what concerns lying quality and quantity, it appears that group size particularly influences how safe the horse feels.

Welfare assessments revealed all horses enjoyed good health and welfare condition during both housing routines and highlights that the ABMs included in the AWIN welfare assessment protocol are not meaningful when a small stressor is applied in the everyday routine of horses. Despite horses spent a significant amount of time in the paddock together with other horses as part of their ordinary routine, where they could possibly be injured by other subjects (25), such injuries were not observed in our study. It appears that the main causes of these types of injuries, such as lack of space and sub-optimal group composition that leads to fights (26) were not present among our groups. One horse did not lay down at all during the observational period. Although adult horses require at least 30 min of lying down per day to achieve 3.5–4.5 min of REM sleep and avoid sleep deprivation, which can result in excessive secondary sleepiness, collapse (11), and severe physiological effects (10), it is not uncommon for individual horses to forgo lying down entirely for a few days. Other studies have also documented instances where horses did not lie down during the observed periods (21, 27).

In this case, ABMs collected twice during our study did not indicate that horses would show any specific health-related issue that could explain the reluctance to lie down (e.g., lameness or back pain). The box size was assessed as appropriate, and the horse could easily lie down and get up. It is then possible that the presence of the accelerometer itself may influence the behavior of the horse, however the horse did not show any signs of discomfort in wearing the accelerometer. To test this hypothesis, it could be suggested to monitor the subject who did not exhibit lying behavior for the same period of time without the accelerometer, or increase the monitoring period with the accelerometer, to see if they actually show reluctance to lie down.

Looking at the lying results, the small differences between the duration of lying in the paddock compared to the indoor box may be attributed to several factors. One possible explanation is that the larger space available in the paddock could promote greater freedom of movement and comfort, potentially encouraging lying behavior (5, 12, 28). Furthermore, the open view provided by the paddock, as well as social companions present, may have created a greater sense of security for the horses, reducing stress and enabling them to rest more comfortably (5). Another possible explanation is the variation in ground materials, as previous studies highlight the importance of bedding for lying behavior. Horses generally prefer to rest and sleep on softer surfaces that provide greater comfort (12, 29). Specifically, wood shavings, the bedding material used in our study, have been reported to be less preferred compared to other options, such as straw, with bedding depth also playing a significant role (29, 30). Furthermore, considering that equine sleep appears to be closely associated with environmental seasonal fluctuations with higher temperature lowering the time spent sleeping, we might think that being in an enclosed environment with the high temperatures recorded at the time of data collection (June 2024) may have affected the duration of sleep itself. Our results are in line with what was found by Chaplin and Gretgrix (21) who also reported that changing the housing conditions (i.e., paddock, fully stabled, partly stabled, and yard) did not significantly affect the time horses spent lying, as well as the number or the duration of lying bouts. All horses were subjected to the same feeding management in both routines, which may have contributed to the lack of differences observed.

While comparing working and non-working days, spending the night in the paddock after work appears to promote increased lying behavior in horses, likely due to the larger space, natural ground surfaces, and greater environmental enrichment compared to confined indoor stalls (5, 12, 28). Also, access to a more natural environment (31) may help reduce stress and fatigue accumulated during work, further supporting the need for adequate recovery and facilitating restorative rest. Grouping horses in paddock during ordinary housing routine revealed the importance of the social group for fostering lying behavior. Among horses, it is widely recognized that living in social groups promotes positive psychological and physical welfare, with social support (i.e., the benefits provided by social partners) enhancing an individual's ability to cope with challenges (32). Additionally, animals that live in groups often present some type of behavioral synchronization (33). In prey animals, behavioral synchronization helps reduce predation risk, while group living also decreases everyone's likelihood of being caught (33). That is why in horses, complete synchronicity of the group is almost never observed while lying or standing resting (34). To ensure safety, individuals often alternate between lying down and standing, creating a rotational vigilance system that helps protect the group (14, 35). This allows individuals to spend more time lying down, sleeping, and resting. Such patterns were clear in our group of four horses, where three horses synchronized their lying behavior, while the fourth horse did not lie down. In contrast, this behavior was less clear in pair of individuals, suggesting that differences in group sizes can probably play a significant role in both synchronization and the duration of lying. However, it is important to note that the observed increase in lying behavior was confounded by several factors, including outdoor access, limited number of groups of the same size, a different substrate for lying, and the presence of social company, therefore drawing definitive conclusions about the specific role of a particular parameter alone is not warranted.

Regarding the hour of the day at which horses lay down most frequently, the results are mainly consistent with reports from previous studies (13, 21, 36). Despite the time of the day when horses decided to lying down seem to differ for every subject between the two routines, considering the group numerosity it has been found that, interestingly, on both working and non-working days, horses from groups of 3 subjects frequently lay down during the day, contradicting previous reports that horses rarely do so outside their usual resting periods (13, 36). Additionally, since ordinary housing required staying in the box during the day, it is remarkable that some horses often lay down indoors (36).

## Conclusions

It appears that, as long as an adequate space for both lying (sternal and lateral) and getting up is provided and sociality is granted thanks to at least visual access to conspecifics, a sudden change in housing routine does not significantly affect lying behavior or animal-based welfare measures. This shows that, for horses, it is possible to show natural behavior such as lying in both housing routines if some species-specific needs are respected.

The use of non-invasive devices such as triaxial accelerometers for continuous monitoring over extended periods has proven to be an effective method for observing behaviors such as lying, including its frequency and duration at the individual level. These devices are also well-suited for use in outdoor environments, making them valuable tools for research aimed at enhancing the welfare assessment of horses, including positive behaviors. Further studies are suggested to better understand the role that group size and social relationships can play for behavioral synchronization of lying in horses as well as acquire more knowledge about the role of restorative rest after a period of work.

## Data Availability

The raw data supporting the conclusions of this article will be made available by the authors, without undue reservation.
