# Supplementary material for: Exploring the impact of housing routine on lying behavior in horses measured with triaxial accelerometer
Source: Front Vet Sci. 2025 May 12;12:1572051. doi: 10.3389/fvets.2025.1572051 (PMC12104677; doi:10.3389/fvets.2025.1572051)
Supplement: Supplementary file 1 [file Table_1.docx]

Table S1. Scoring system for animal-based measures as reported in AWIN Welfare Assessment protocol for horses.

| **Animal-based measure** | **Score** |
| --- | --- |
| Body Condition Score | Score 1-5, with score 3 considered as appropriate |
| Horse Grimace Scale (HGS) | Score 0-12, with scores <3 considered as appropriate |
| Stereotypies | Present - Absent |
| Voluntary animal  approach test | Positive - Negative - No interest, with score “positive” considered as appropriate |
| Avoidance distance test | Avoidance - No avoidance |
| Hair coat condition | Healthy – Unhealthy |
| Abnormal breathing | Present - Absent |
| Cough | Present - Absent |
| Swollen joints | Present - Absent |
| Hoof neglect | Present - Absent |
| Discharges | Present - Absent |
| Prolapse | Present - Absent |
| Integument alterations | Absence of alopecic areas, skin lesions, deep wounds, swellings was considered appropriate |
| Bite injuries (social) | Present - Absent |
| Injuries related to management | Present - Absent |
