# Supplementary material for: Exploring the impact of housing routine on lying behavior in horses measured with triaxial accelerometer
Source: Front Vet Sci. 2025 May 12;12:1572051. doi: 10.3389/fvets.2025.1572051 (PMC12104677; doi:10.3389/fvets.2025.1572051)
Supplement: Supplementary file 2 [file Table_2.docx]

Table S2. Group composition during ordinary housing routine.

| Horse ID | Sex | Age | Group size | Group members IDs |
| --- | --- | --- | --- | --- |
| 1 | Gelding | 19 | 4 | 1, 2, 3, 4 |
| 2 | Gelding | 19 |  |  |
| 3 | Gelding | 10 |  |  |
| 4 | Mare | 11 |  |  |
| 5 | Mare | 12 | 3 | 5, 6, 7 |
| 6 | Mare | 11 |  |  |
| 7 | Mare | 16 |  |  |
| 8 | Mare | 20 | 2 | 8, 9 |
| 9 | Mare | 15 |  |  |
| 10 | Gelding | 15 | 1 | 10 |
